# A Randomized, Placebo-Controlled, Single-Center, Crossover Study to Evaluate the Effects of Pre-Meal Whey Protein Microgel on Post-Prandial Glucometabolic and Amino Acid Response in People with Type 2 Diabetes and Overweight or Obesity

**DOI:** 10.3390/metabo15010061

**Published:** 2025-01-16

**Authors:** Ian J Neeland, Luiz H de Gregório, Roberto Zagury, Bo Ahrén, Joel Neutel, Christian Darimont, John Corthesy, Yohan Grzywinski, Emilie Perrin, Maximilian von Eynatten, Odd Erik Johansen

**Affiliations:** 1 Harrington Heart and Vascular Institute, Division of Cardiovascular Medicine, University Hospitals Cleveland Medical Center, Cleveland, OH 44106, USA and Department of Medicine, Case Western Reserve University School of Medicine, Cleveland, OH 44106, USA; ian.neeland@uhhospitals.org; 2 IBPClin, Rio de Janeiro 20241-180, Brazil; luiz.gregorio@careaccess.com; 3 Human Performance Lab, Rio de Janeiro 22271-070, Brazil; roberto.zagury@gmail.com; 4 Department of Clinical Sciences, Lund University, 221 00 Lund, Sweden; bo.ahren@med.lu.se; 5 Orange County Research Center, Tustin, CA 92780, USA; neutelmd@ocresearchcenter.com; 6 Societe de Produits Nestlé, 1000 Lausanne, Switzerland; christian.darimont@rdls.nestle.com (C.D.); john.corthesy@rd.nestle.com (J.C.); yohan.grzywinski1@rd.nestle.com (Y.G.); 7 SOCAR Research SA,1260 Nyon, Switzerland; 8 Nestlé Health Science, 1000 Lausanne, Switzerland; maximilian.eynatten@nestle.com

**Keywords:** type 2 diabetes, obesity, GLP-1, whey protein micelles, insulin, amino acid

## Abstract

**Purpose**: Whey protein (WP) consumption prior to a meal curbs appetite and reduces postprandial glucose (PPG) through stimulating endogenous GLP-1 secretion and insulin. **Methods**: We assessed the metabolic effects of a concentrated WP, using a new micelle-technology (WPM), in people with type 2 diabetes (T2D) and overweight or obesity (NCT04639726). In a randomized-crossover design, participants performed two 240 min lunch meal (622 kcal) tests 7 ± 4 days apart. After an overnight fast and a standardized breakfast, 10 g (125 mL) WPM (40 kcal) or placebo (125 mL water, 0 kcal) was consumed 15 min ahead of the mixed-nutrient meal. Effects on PPG (primary endpoint), insulin, GLP-1, and branched-chain amino acids (BCAAs) were evaluated with frequent blood sampling. Changes in incremental areas under the concentration curve (iAUC) were compared using a mixed model. **Results**: Twenty-six individuals (14 females, mean ± SD age 62.0 ± 8.3 years, HbA1c 58 ± 12 mmol/mol/7.5 ± 1.1%, BMI 29.2 ± 4.8 kg/m^2^) completed both tests. WPM significantly reduced PPG iAUC_0–2h_ by 22% (*p* = 0.028), and iAUC_0–3h_ numerically by −18% (*p* = 0.090) vs. placebo. WPM also increased insulin iAUC_0–1h_ by 61% (*p* < 0.001), and iAUC_0–3h_ by 30% (*p* = 0.004), respectively. Total GLP-1 iAUC_0–2h_ was enhanced by 66% (*p* < 0.001). Postprandial plasma BCAA patterns were characterized by a rapid increase and larger iAUC_0–2h_ (all *p* < 0.001) after WPM. No adverse events were ascribed to consuming WPM. **Conclusions**: A 125 mL pre-meal drink containing just 10 g WPM before a mixed meal reduced PPG and increased insulin, GLP-1, and BCAAs. WPM may therefore serve as a metabolic modulator in people with T2D living with overweight or obesity.

## 1. Introduction

Whey proteins (WPs) are found in dairy products, and are rich in branched-chain amino acids (BCAA), i.e., leucine, isoleucine, valine, other AAs, and bioactive peptides, e.g., α—lactalbumin and β—lactoglobulin, that stimulate the secretion of incretin peptides, like glucagon like-peptide 1 (GLP-1) and glucose-dependent insulinotropic polypeptide (GIP) [1]. The downstream effect of stimulating incretin peptides has been associated with important clinical metabolic benefits [2,3], and is mediated via augmented release of insulin in a glucose-dependent manner, slowing of the rate of gastric emptying (GE) mediated through vagal afferents, hence regulating the gastrointestinal (GI) transit of food, and suppressing appetite [4,5,6,7]. WP also has a robust ability to stimulate muscle protein synthesis due to its favorable amino acid composition (high in essential AAs, in particular leucine) and rapid digestibility [8], and thus also may have a favorable effect on body composition [9].

The most notable early metabolic clinical effect observed by consuming WP, in particular as a pre-meal intervention, is a lowering of post-prandial glucose (PPG) excursion, observed both in people with normal glucose metabolism [10], as well as in people with prediabetes [11] or type 2 diabetes (T2D) [12]. Reducing PPG contributes to reducing the overall glycemic burden. This is particularly important in prediabetes and early stages of T2D, where the relative contribution of PPG, as compared with fasting glucose, to overall glucose burden reflected by HbA1c level, is more important [13]. WP could therefore be an adjunct to any intervention to support managing glucose levels, e.g., through lifestyle, or pharmaceutical interventions. However, routine nutraceutical clinical use of WP as a pre-meal intervention has traditionally been limited by requiring a relatively high WP dose, contributing to a high caloric content (each gram of protein provides ~4 kilocalories) [14], and a requirement to ingest it well in advance of a meal in order to induce the relevant downstream metabolic responses discussed above. To illustrate, studies that presented 50 g or 25 g of WP 30 min before a nutrient challenge [15,16] both induced a reduction in PPG and an augmented GLP-1 response, whereas 20 g [17] or 15 g did so to a lesser degree or not at all [18]. New technologies that could address these practical hurdles, e.g., shorten the time of ingestion before a meal and lower the caloric impact, would be most welcome. One such new technology that could enable both use of a lower dose, as well allow ingestion closer to the meal, is a new WP formulation (WP microgel [WMP]) [19], developed as a specific form of WP aggregate, delivered in a low-dose (10 g), but highly concentrated, ready-to-drink (RTD) solution. In healthy individuals, this novel formulation has been suggested to modulate PPG [20]. Herein we tested if 10 g WPM ingested 15 min before a mixed-nutrient meal could provide clinically meaningful effects on glucometabolic parameters, aminoacidaemia, and GE, in individuals with T2D living with overweight or obesity.

## 2. Materials and Methods

### 2.1. Study Design and Patients

This was a mechanistic, randomized, investigator-blinded, placebo-controlled, single-center exploratory crossover study (ClinicalTrials.gov: NCT04639726) that recruited adults (men and women age ≥ 18 years) with T2D that presently were not taking any medications for glycemic management, or were on stable metformin monotherapy 1–3 g/day. This study was designed to evaluate the effects of consumption of WPM (10 g) compared with placebo (water) as a 125 mL pre-meal RTD solution on PP metabolic parameters and GE (Supplementary Figure S1). The study protocol was approved by the Institutional Review Board of Orange County Research Center, USA (wcg Aspireirb, IRB tracking number 20202308, approved 9 September 2020), and the study was carried out in compliance with the Harmonized Tripartite Guideline for Good Clinical Practice from the International Conference on Harmonisation [21] and the Declaration of Helsinki [22]. Participants provided written, informed consent.

In addition to a diagnosis of T2D (from medical history, or by HbA1c 6.5–10.0%), the other key inclusion criteria, due to frequent blood sampling, were haematocrit ≥ 34/40% for females/males, and hemoglobin ≥11.0/13.5 g/dL for females/males, respectively. Key exclusion criteria were fasting plasma glucose >220 mg/dl, estimated glomerular filtration rate (eGFR) < 60 mL/min/1.73 m^2^, body mass index (BMI) > 40 kg/m^2^ (with no lower BMI cut-off), and ongoing or recent (i.e., <3 month) treatment with any oral or injectable glucose-lowering medications other than metformin. The full list of inclusion and exclusion criteria is provided in the Supplementary Table S1.

### 2.2. Investigational Products

The pre-meal drink of 10 g of WP (40 kcal) was prepared with a novel technology aimed at increasing the WP concentration in a liquid matrix that did not gel, using a proprietary micelle-technology by generating microgels (WPM) [19]. The technology behind the formation of the novel concentrated WPM involves several steps, including heat treatment, concentration by conventional evaporation, and pH adjustment of native WP [19]. The active product contained 86.8% WP, 10.7% caseins, and 2.5% of casein glycomacropeptide or other proteins. The WPM was produced from a native whey protein isolate (Pronativ9, Lactalis Ingredients, France) [23]. The WPM was fully diluted in 125 mL water, without any evidence of gelling, and produced a palatable, RTD beverage, that in industrial production has a shelf life of 12 months and is stable at both room temperature and chilled environmental conditions (available on the US market under the BOOST^®^ brand). The matching placebo was 125 mL of water. The key considerations for selecting water as a comparator were based on its ease of preparing an iso-volumetric comparator for the RTD protein beverage, and its metabolically inert properties, which was important for the study objective of assessing postprandial effects.

Except for the unmasked pharmacist who prepared the investigational product for consumption, the investigator, all site study personnel, and participants remained blinded to the treatment, and both the active and the placebo (i.e., water) pre-meal drink were provided in forms of identical appearance.

### 2.3. Study Procedures

All participants attended the clinic on three separate occasions (screening, visit 1, and visit 2), where visit 1 and 2 represented the intervention visits, which were separated by 7 ± 4 days of wash-out. Both interventions were to be ingested as an RTD beverage, 15 min ahead of a mixed lunch meal consisting of 250 g of pizza (McCain BBQ Meatlovers Family Pizza) delivering 2600 kJ/622 kcal (29.0 g protein, 24.2 g total fat (12.6 g saturated fat), 68.8 g carbohydrates (14 g sugars), and 1026 mg sodium). The pizza meal was ingested within 15 min with 150 mL of water.

To standardize the physiologic conditions ahead of the lunch meal to enhance the interpretability of results, participants were encouraged to consume similar evening meals prior to clinic visits, and were required to fast overnight (at least 10 h) prior to site-arrival in the morning, whereupon they were served a standardized breakfast, selected based on general acceptance level and ease of availability, consisting of one portion (27 g) of ready-to-eat breakfast cereals (Cheerio’s, Nestlé, Rosslyn, VA, USA, 98.6 kcal [2.2 g protein, 0.8 g fat, 20.0 g carbohydrates]), with 250 mL milk (2% fat, 129 kcal [8.5 g protein, 5.1 g fat, 13.0 g carbohydrates]), to be ingested within 15 min. Thereafter, four hours elapsed, during which only water was allowed to be consumed (ad libitum for the first three hours), and only light exercise (i.e., walking) allowed, until the pre-meal investigational RTD beverage was taken. The pizza meal was served 15 min after this, alongside 1 g of acetaminophen (for evaluate GE), with subsequent frequent blood sampling for glucometabolic and GE assessment (Supplementary Figure S2). All blood samples were taken from subjects by venepuncture or cannulation, and serum and plasma were prepared using standard procedures.

### 2.4. Primary Exploratory Endpoint

The study hypothesis was that a low dose of WPM given 15 min before a standardized mixed meal would lower the PP blood glucose levels compared to placebo in subjects with T2D; thus, the primary endpoint was to assess effects on PP glucose over 4 h, which, relative to the first post-meal measurement, was assessed at −45, −30, 0 (i.e., first post-meal assessment), 15, 30, 45, 60, 90, 120, 150, 180, and 240 min. This was an exploratory study with both pre and post hoc analyses defined (Supplementary Table S2), where the primary exploratory analysis was defined as the 3 h incremental area under the curve (iAUC) for sample size estimation. Glucose was analyzed in EDTA-plasma (Cobas c501 Systems, Roche Diagnostics, Indianapolis, IN, USA), and the baseline glucose value was calculated as the mean of the −45 min and −30 min assessments.

### 2.5. Other Metabolic Endpoints

Interval blood samples up to four hours were also collected for insulin (serum, Immulite 2000 Analyzer, Consolidated Medical Bio-Analysis, Inc., Cypress, CA, USA), and glucagon (EDTA plasma, enzyme immunoassay, Consolidated Medical Bio-Analysis, Inc., Cypress, CA, USA). The following parameters were only assessed up to two hours (−30 min [baseline values], 0, 30, 60, 90, and 120 min): total endogenous GLP-1 (EDTA-plasma, Merck Millipore, MA, USA), PYY (EDTA-plasma, Merck Millipore, MA, USA), total GIP (EDTA-plasma, Merck Millipore, MA, USA), CCK (plasma, radioimmunoassay, LabCorp, Burlington, NC, USA), active ghrelin (EDTA-plasma, radioimmunoassay, Linco Research, St Charles, MO, USA), triglycerides (enzymatic colorimetric assay, Consolidated Medical Bio-Analysis, Inc., Cypress, CA, USA), and amino acids (high-performance liquid chromatography/mass spectrometry, Nestlé Research, Lausanne, Switzerland) (Supplementary Figure S2).

### 2.6. GE Assessment

GE is the rate-limiting step for the appearance of acetaminophen (N-acetyl-p-aminophenol, paracetamol) within blood [24], since it is poorly absorbed by the stomach but rapidly absorbed within the small intestine. AUC, often assessed during the first 60 min (max concentrations typically occurs ~30–60 min post-ingestion), is used as a marker of GE rates, although two, three, or four hours are also frequently reported. Herein we assessed both short-term (0–1 h) as well as total GE (0–4 h) in interval blood samples (Sannova Analytical, Somerset, NJ, USA) following ingestion of 150 mL of water containing 1 g dissolved liquid acetaminophen (MAJOR Pharmaceuticals, Livonia, MI, USA).

### 2.7. Safety and Adverse Events (AEs)

General safety laboratory tests were drawn at the screening visit to determine eligibility. Occurrence of AEs was proactively assessed by queries at all visits post screening, and all AEs (spontaneously reported or enquired, as well as those observed) during the course of the study (from visit 1 post-dose through the visit 2 [Day 7 ± 4]) were captured, and were summarized descriptively. AEs were coded using Medical Dictionary for Regulatory Activities (MedDRA), Version 23.0.

### 2.8. Statistical Methods, Randomization, and Sample Size Considerations

Randomization was preplanned to occur in a 1:1 ratio to either sequence AB or BA on visit day 1 according to the crossover design, and was stratified by metformin use at baseline. Patients assigned to sequence AB received the WPM beverage at visit 1 and placebo at visit 2, whereas patients assigned to the BA sequence received placebo at visit 1 and the WPM beverage at visit 2.

The study hypothesis was that a low dose of WPM given 15 min before a standardized mixed meal would lower the PP blood glucose levels compared to placebo in individuals with T2D. In order to assess and characterize both early and late effects of the intervention, we assessed this over 4 h as described above. The difference between WPM and placebo was assessed by comparing changes in iAUC between the two interventions over different time periods using one-way analysis of variance (ANOVA). Although this was an exploratory study, we derived the sample size based on assumptions that we could demonstrate a difference of 15% in the iAUC of PP glycemic excursion between WPM and placebo over a 3 h period. We used a coefficient of variation (CV) approach, and based on two previous studies assessing PP glucose trajectories [24,25] reporting this to be 0.41 and 0.50, we assumed a CV of 0.46. Given a within-patient correlation observed from a different study of 0.88 (data on file), we conservatively assumed this to be 0.85. With a one-way repeated measures (ANOVA) analysis, 25 participant completers would then be required to show a statistically significant difference of 15% in iAUC 0–3 h for glucose, at a α-level of 0.05 with a power of 80%.

Biomarkers were analyzed with a positive iAUC approach, with the exception of ghrelin, where a reduction in levels was expected following nutrient challenge; hence, they were analyzed with an iAUC below the baseline value approach. Analysis of gastric emptying was performed with a total AUC (tAUC) approach. Relative difference between treatment groups was calculated in % as estimated treatment difference/estimated mean for placebo ×100%. We also assessed if there were differences in both the time to reach maximum concentration levels of the biomarkers (T_max_), and in maximum levels of the biomarkers reached (C_max_).

### 2.9. Analysis

Depending on the distribution of data, patient characteristics were described using mean ± standard deviation, mean ± standard error, or mean (min, max) for continuous variables and proportions for categorical variables. Descriptive data for graphical presentation are shown as mean (standard error).

Comparisons were performed by comparing mean changes in iAUC between the intervention and placebo groups over the different time periods using ANOVA (mixed model) with intervention arm and period (period 1 and period 2) as fixed effects and subject as random effect. Results are expressed as effect estimates (LSmeans) with a 95% confidence interval (CI). T_max_ and C_max_ differences were assessed by the same model, except for between AAs, where the Friedman test was used. A two-sided nominal *p*-value < 0.05 was conventionally considered significant, and we did not correct for multiple testing. Estimated GFR (eGFR) was derived by the Modification of Diet in Renal Disease (MDRD) study equation.

## 3. Results

### 3.1. Participant Characteristics

In total, 26 individuals (14 females) were recruited (September–October 2020) and completed two sequences of treatment according to the randomization scheme. There were no drop-outs (consort diagram in Supplementary Figure S3). Overall, the mean ± SD age was 62.0 ± 8.3 years, HbA1c 58 ± 12 mmol/mol/7.5 ± 1.1%, and eGFR 99.1 ± 24.7 mL/min/1.73 m^2^. Mean BMI was 29.2 ± 4.8 kg/m^2^ while mean weight and waist circumference were 82.9 ± 15.0 kg and 101.3 ± 12.7 cm, respectively. Nineteen (73%) participants were using metformin during the study (Table 1).

### 3.2. Effects on Glucose, Insulin, and Glucagon

**Figure 1:**
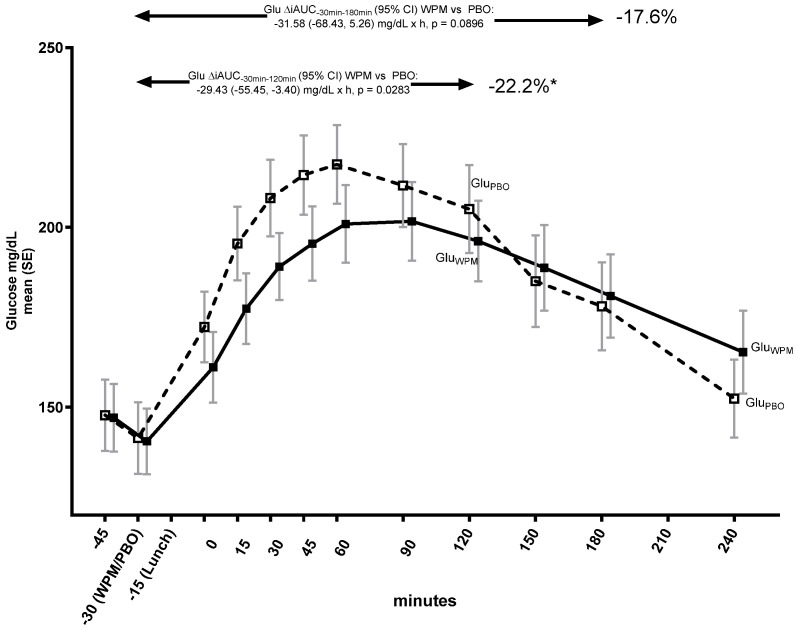
Glucose trajectory over 4 h following pre-meal consumption of 10 g ultra-concentrated whey protein microgel or placebo to a mixed lunch meal in people with type 2 diabetes mellitus. Abbreviations: Glu—glucose, iAUC—incremental area under the curve, PBO—placebo, WPM, whey protein microgel, SE—standard error, h—hours. *: *p* < 0.05.

The pre-meal WPM drink significantly altered the early PP glucose trajectory (Figure 1), and reduced the 2 h iAUC by 22.2% (mean [95% CI] ΔiAUC_−30min–120min_ WPM vs. placebo −29.4 [−55.5, −3.4] mg/dL×h, *p* = 0.0283), whereas the 3 h iAUC was numerically reduced by 17.6% (ΔiAUC_−30min–180min_ −31.6 [−68.4, 5.3], *p* = 0.0896) (Figure 1).

**Figure 2:**
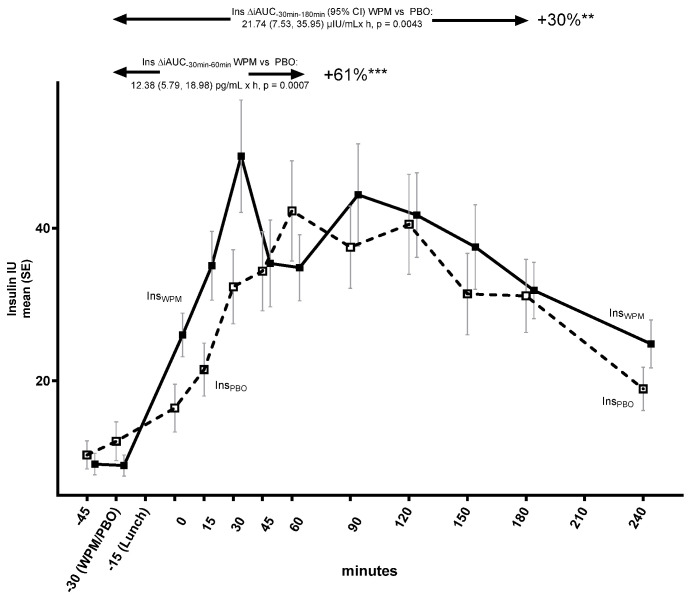
Insulin trajectory over 4 h following pre-meal consumption of 10 g ultra-concentrated whey protein microgel or placebo to a mixed lunch meal in people with type 2 diabetes mellitus. Abbreviations: Ins—insulin, iAUC—incremental area under the curve, PBO—placebo, WPM, whey protein microgel, SE—standard error, h—hours. **, ***: *p* < 0.01, 0.001.

There was no difference in achieved maximum glucose levels (mean [95% CI] C_max_ WPM: 215.85 [193.26, 238.43] mg/dL; placebo: 225.38 [202.80, 247.97] mg/dL; ΔC_max_ WPM vs. placebo: −9.54 [−28.20, 9.12], *p* = 0.3019), but the time to reach it (T_max_) was significantly longer with WPM (104.4 [85.9, 122.9] min) relative to the placebo (59.4 [40.9, 77.9]); ΔT_max_ WPM vs. placebo: 45.0 (21.9, 68.1) min (*p* = 0.0005), indicating a less rapid PPG excursion with WPM compared with the placebo; see also Supplementary Table S3 for the C_max_ and T_max_ of the glucometabolic and gastric emptying parameters assessed up to 4 h in blood for WPM versus placebo. Both the insulin (Figure 2), and glucagon (Supplementary Figure S3) PP trajectories were also modulated with WPM.

The increase in insulin was significantly enhanced, with an indication of restoration of the bi-phasic insulin secretion pattern vs. placebo, with a 61% increased insulin release during the first 60 min (ΔiAUC_−30min–60min_ WPM vs. placebo 12.4 (5.8, 19.0) μIU/mL × h, *p* = 0.0007), and a 30% increase over 3 h (ΔiAUC_−30min–180min_ WPM vs. placebo 21.7 (7.5, 36.0) µIU/mL × h, *p* = 0.0043). There were no differences in insulin C_max_ or T_max_ between the two groups. A significant increase in glucagon secretion was also observed with the WPM, with a similar prominent early 64.5% increase during the 1st hour (ΔiAUC_−30min–60min_ WPM vs. placebo: 14.2 [5.1, 23.3], *p* = 0.0037), as well as a sustained 50.0% increase over 3 h (ΔiAUC_−30min–180min_ WPM vs. placebo: 30.0 [10.5, 49.6], *p* = 0.0042). C_max_ and T_max_ for the glucagon levels were directionally higher, and shorter with WPM (Supplementary Table S3), respectively, but did not reach statistical significance (ΔC_max_ WPM vs. placebo: 14.5 [−5.3, 34.3] pg/mL, *p* = 0.1441; ΔT_max_ WPM vs. placebo −24.8 [−64.9, 15.3] min, *p* = 0.2194).

### 3.3. Effects on Gut Hormones and Triglycerides

**Figure 3:**
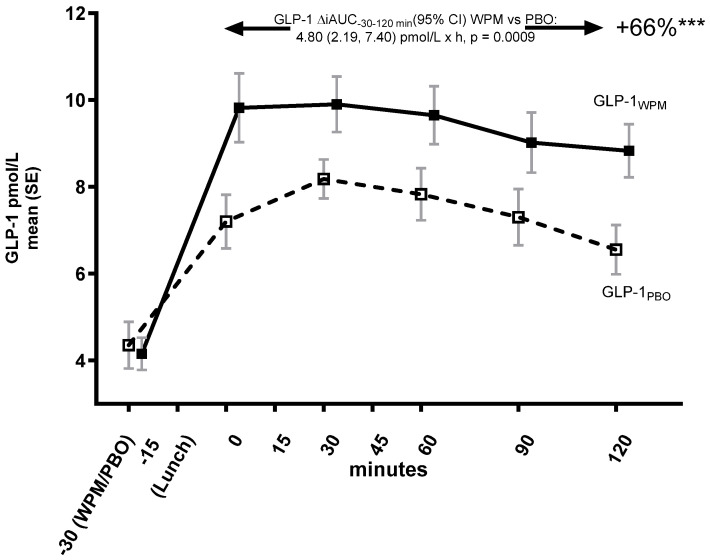
Glucagon-like peptide 1 trajectory over 2 h following pre-meal consumption of ultra-concentrated whey protein microgel or placebo to a mixed lunch meal in people with type 2 diabetes mellitus. Abbreviations: GLP-1—Glucagon-like peptide 1, iAUC—incremental area under the curve, PBO—placebo, WPM, whey protein microgel, SE—standard error, h—hours. ***: *p* < 0.0001.

A 66% increase in GLP-1 iAUC_−30min–120min_ (Figure 3) was observed (between-group difference: 4.8 [2.2, 7.4] pmol/L × h, *p* = 0.0009), with differences between treatment groups occurring early and the maximum concentration achieved being higher (WPM: 12.0 [10.6, 13.5]; placebo: 9.2 [7.7, 10.7]; between group difference 2.8 (1.1, 4.6), *p* = 0.0032).

Responses for GIP, CCK, and PYY (Supplementary Figure S5A–C) were similar between WPM and placebo (between-group difference in iAUC_−30min–120min_ 8.5 [−15.3, 32.3] pmol/L × h, *p* = 0.4668, −26.2 [−71.9, 19.5] pg/mL × h, *p* = 0.2475, and, 5.2 [−1.1, 11.6] pmol/L × h, *p* = 0.1035, respectively). For ghrelin (Supplementary Figure S5D), the negative iAUC_−30min–120min_ with the WPM pre-meal drink was −283.3 (−456.8, −109.8) pg/mL × h, which was numerically lower than with the placebo (−130.2 [−295.4, 35.0] pg/mL × h); however, no significant between-group difference was observed (−153.1 [−393.6, 87.4] pg/mL × h, *p* = 0.1956). There were complete samples available for analysis of all gut hormones except ghrelin, where only 14/26 participants had valid values in the WPM group and 15/26 participants in the placebo group, owing to an analysis resulting in “below limit of quantification”. There were no notable differences in triglycerides between WPM and the placebo (Supplementary Figure S5E), and no notable differences in C_max_ and T_max_ between WPM and the placebo were observed for GIP, CCK, PYY, ghrelin, or triglycerides (Supplementary Table S3).

### 3.4. Effects on Amino Acids

**Figure 4:**
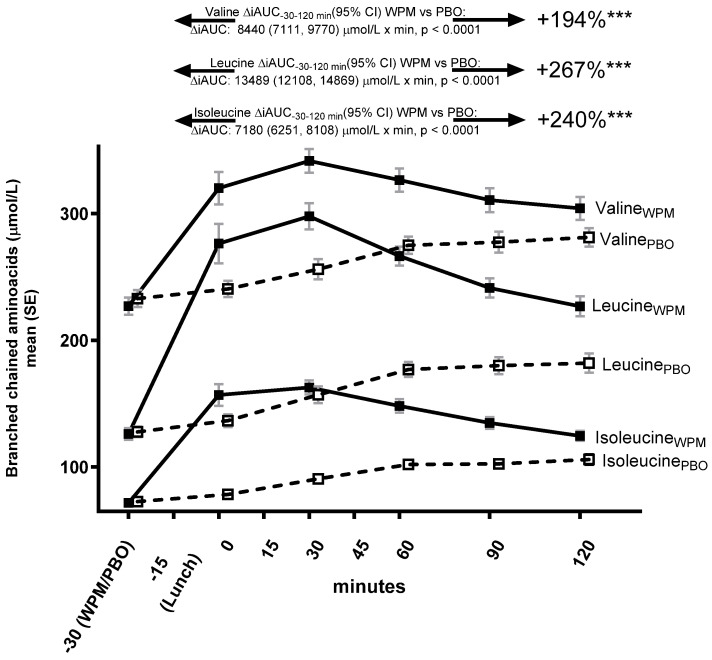
Valine, leucine, and isoleucine trajectories over 2 h following pre-meal consumption of ultra-concentrated whey protein microgel or placebo to a mixed lunch meal in people with type 2 diabetes mellitus. Abbreviations: iAUC—incremental area under the curve, PBO—placebo, WPM, whey protein microgel, SE—standard error. ***: *p* < 0.0001.

A total of 29 plasma amino acid profiles were assessed in 25 participants with available samples. The 10 g WPM as a pre-meal drink followed by a mixed lunch meal induced a rapid plasma increase, and significantly higher bioavailability of all BCAAs in people with T2D (Figure 4) compared with the placebo.

For leucine, ΔiAUC_−30min–120min_ increased by 267% (13,489 (12,108, 14,869) μmol/L × min, *p* < 0.0001), with a shorter time to maximum (median ΔT_max_ [min, max]: −60 [−120, 0] min, *p* < 0.0001), and peak concentration (mean ΔC_max_ [SD]: 123 [49] umol/L, *p* < 0.0001). A similar pattern, with a 240% increase, was seen for isoleucine for WPM vs. PBO (ΔiAUC_−30–120 min_ [95% CI]: 7180 (6251, 8108) μmol/L × min, *p* < 0.0001) and shorter time to peak and maximum concentrations (ΔT_max_: −60 [−120, 0] min, *p* < 0.0001, ΔC_max_: 68 [3] μmol/L, *p* < 0.0001), as well as for valine (ΔiAUC_−30min–120min_ increased by 194% [8440 (7111, 9770) μmol/L × min, *p* < 0.0001], ΔT_max_: −60 [−120, 30] min, *p* < 0.0001, ΔC_max_: 63 [38] μmol/L, *p* < 0.0001).

Also, several other amino acid profiles followed the same pattern (lysine, methionine, tyrosine, and arginine) (data not displayed). Some AA profiles were characterized by an early increase, which was not sustained over the 2 h period (alanine, aspargine, glutamic acid, histidine, phenylalanine, proline, serine, threonine, aspartic acid, ornithine, and ADMA), whereas for the remainder of the analyzed AAs, there were no differences between the interventions (data not displayed).

### 3.5. Effects on GE

**Figure 5:**
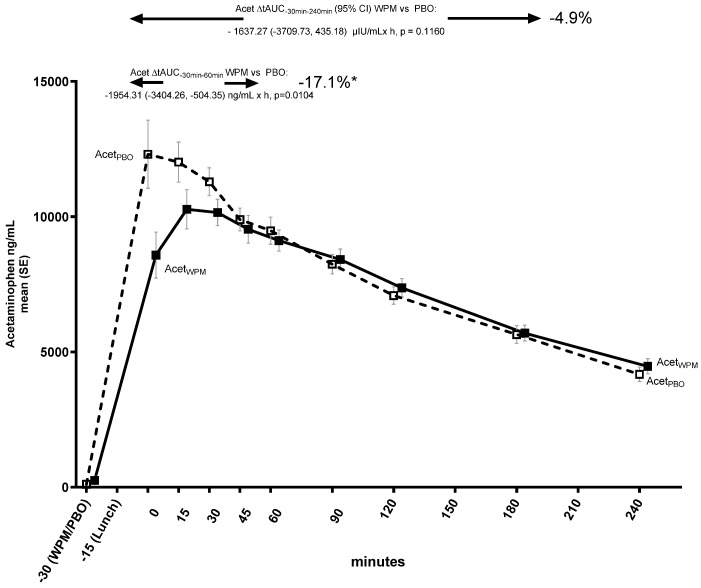
Effect on gastric emptying expressed by mean acetaminophen (paracetamol) trajectory over 4 h following pre-meal consumption of ultra-concentrated whey protein microgel or placebo to a mixed lunch meal in people with type 2 diabetes mellitus. Abbreviations: tAUC—total area under the curve, PBO—placebo, WPM, whey protein microgel, Acet—acetaminophen (paracetamol), SE—standard error, h—hours. *: *p* < 0.05.

There was an early delay of GE (first 30 min) with WPM (Figure 5), with a difference in tAUC_−30min–60min_ WPM vs. placebo of −1954.3 (−3404.3, −504.4) ng/mL × h (*p* = 0.0104). There was also a difference in the maximal concentration of acetaminophen, which was 17.2% lower with WPM compared with the placebo (C_max_ placebo: 14156.5 vs WPM: 11719.5 ng/mL; between group difference: −2437.0 [−4271.0, −603.1] ng/mL, *p* = 0.0113), indicating quantitatively slower and lower GE with WPM (Supplementary Table S3). However, no significant difference in overall GE was seen (tAUC_−30min–240min_ WPM vs. placebo −1637.3 [−3709.7, 435.2] ng/mL × h [*p* = 0.1160]).

### 3.6. Adverse Events

There were no adverse events or serious adverse events reported in this study.

## 4. Discussion

In individuals with T2D and overweight or obesity, an ultra-concentrated RTD pre-meal formulation of a low dose of WP (10 g; 40 kcal) provided as WPM in a 125 mL solution, shortly (15 min) ahead of a lunch meal, reduced PPG, and increased PP insulin and GLP-1 response. BCAA trajectories were also significantly amplified with the WPM, and there was also an early delay in GE (mimicking physiological effects of GLP-1). These results are important from several perspectives. First, although it has been known for a long time [1] that WP can modulate glucometabolic response, these beneficial modulations have traditionally been seen with a relatively high amount (typically 25–50 g) and caloric dose of WPs [15,16,25,26,27], or only if the WP has been taken well in advance (typically 30–60 min) of the meal [15,16]. The former is a nutraceutical barrier for widespread use, as with multiple daily consumptions, this could add significantly to the caloric burden of the individual; hence, it not only represents a practical limitation, but also could be an issue from a weight management perspective [1].

The literature to date for traditional WP solutions suggests that there is a clear dose–response with the amount of WP ingested [28], which makes the observation with a low-dose, ultra-concentrated WP formulation used in the present study notable. Another fairly recent study involving 12 lean males, and 12 males with obesity but without T2D, also studied a relatively small amount of WP (15 g [100 kcal]), using a hydrolyzed WP ingredient, provided as an RTD solution and taken 10 min before a mixed-nutrient meal [29]. In this study, the WP intervention reduced the PPG AUC at 60 min by 13% and 18.2%, respectively, in the lean participants and the participants with obesity, which is less than what was observed in the present study in people with T2D. Furthermore, in contrast to our findings, there were no sustained effects on GLP-1 or on insulin, and data suggested in fact that in lean participants, WP induced a reduced GLP-1 degradation rather than increased GLP-1 secretion [29]. Another study, also assessing a smaller amount of WP (20 g, 80 kcal) or placebo as a pre-meal intervention, reported a lack of effect on glycaemia when consumed 15 min before a fat-rich meal, and as observed in the former study, there was no effect on GLP-1 [17]. In the present study, a robust and sustained effect on both GLP-1 and insulin was observed with a small dose of WP (10 g, 40 kcal), as well as an enhancement of plasma BCAAs, and early delayed GE.

The timing consideration of WP pre-meal consumption is another important practical question. Several studies have attempted to address this, and even attempted to close the gap completely; one involved men with T2D, where 15 g (68 kcal) of intact WP was given at the same time as the meal [18]. In that study, WP improved both PP glycemia (assessed with a continuous glucose monitoring device) and had an effect on insulin release, but failed to have an effect on GLP-1 levels.

We speculate that the low-dose WP in a WPM formulation used in the present study was able to generate a more robust glucometabolic response, including an effect on GLP-1, which may be related to the micelle-technology applied that might enable a higher bioavailability of AAs, in particular the BCAAs, and potentially other bioactive peptides [1,30,31,32]. In this regard, interesting insights stem from preclinical work where it is reported that some AAs stimulate GLP-1 secretion only from the intestinal lumen, whereas others exclusively stimulate secretion from the vascular side, indicating that AA-stimulated GLP-1 secretion involves both apical and basolateral (postabsorptive) sensing mechanisms [33]. Since BCAAs have predominantly been seen as having a “flushing” effect, this may suggest that their immediate bioavailability is important [33]. Our hypothesis also finds some support from a human study that compared iso-energetic liquid casein with whey protein preloads, where they related an observed difference in satiety to a larger post-absorptive increase in plasma AA, in particular BCAAs [34]. We believe that these unique features of the WPM formulation make it more feasible to ingest the WP as a pre-meal shot closer to the meal, than what previously has been studied with more regular WP formulations. Whether simultaneous ingestion of the WPM at the time of the meal would demonstrate the same metabolic effects has not been studied with this WPM formulation in people with T2D, and would need further investigation.

This study found a delay in early GE with a directionally consistent (but not statistically significant) effect on ghrelin, as well as a significant difference in PPG Tmax, which is notable in the context of WP being suggested to have a more profound effect on reducing appetite and decreasing ad libitum energy intake at a subsequent meal compared with the other protein meals [34,35]; thus, it possibly has meal-regulating potential for weight loss in overweight or obese individuals, since GE is also associated with sensations of appetite and rapid GE is associated with increased appetite [36]. Further prospective studies, involving the use of validated questionnaires, are needed with this WPM, preferably with multiple dosing.

Limitations of this study include the single-center design, only acute dosing, a relatively modest study population, and the majority of the study population being Caucasians. However, given that previous studies do not suggest a huge difference in WP response across baseline characteristics, we believe that this study’s findings are generalizable outside of the particular study population. We also used the acetaminophen test for GE, which some argue is inferior to other methods like scintigraphy; however, studies have demonstrated that it is a reasonable proxy [24] with less risk. Also, we did not measure intact GLP-1 levels, which would be required to establish whether WPM alters GLP-1 degradation in addition to GLP-1 secretion. Finally, we did a number of post hoc analyses, where multiple testing was not corrected for, which may increase the chance of type II error, and further confirmatory studies are required.

## 5. Conclusions

An ultra-concentrated 10 g (40 kcal) WP formulation as a WPM, provided as an RTD solution and consumed 15 min ahead of a lunch meal in people with T2D and overweight or obesity, reduced PPG, increased PP insulin, GLP-1, and BCAA response, and induced an early delay in GE. Whether these findings translate into long-term benefits for HbA1c, body weight, satiety, or body composition requires further investigation.

## Figures and Tables

**Table 1:** Baseline characteristics of the 26 participants in the study with type 2 diabetes mellitus and overweight or obesity. *n* (%) or mean (SD).

Parameter/Characteristic	Value
	***n*** **(%)**
Sex (female/male)	14 (54%)/12 (46%)
Age (years)	62.0 (8.3)
Race ^1^	
Asian	3 (11.5%)
Black or African-American	4 (15.4%)
White	19 (73.1%)
Other/not reported	0 (0%)
Ethnicity	
Hispanic or Latino	9 (34.6%)
Not Hispanic or Latino	17 (65.4%)
Medications	
Metformin	19 (73%)
Glimepiride	1 (4%)
Statins	8 (31%)
	**Mean (SD)**
Systolic BP/Diastolic BP (mmHg)	129 (12)/77 (9)
Weight (kg)	82.9 (15.0)
Body Mass Index (BMI) (kg/m^2^)	29.2 (4.8)
Waist circumference (cm)	101.3 (12.7)
HbA1c (%) ^2^	7.5 (1.1)
Fasting plasma glucose (mg/dL) ^3^	139.9 (42.9)
Total cholesterol (mg/dL) ^4^	180.4 (50.0)
Triglycerides (mg/dL) ^5^	159 (62)
eGFR (ml/min/1.73 m^2^) ^6^	99.1 (24.7)

^1^: as identified by participants; ^2^: 58 mmol/mol; Old HbA1c = 0.0915 New + 2.15%; ^3^: 7.8 mmol/L; mg/dL to mmol/L glucose: multiply with 0.0555; ^4^: 4.7 mmol/L; multiply by 0.02586; ^5^: 1.8 mmol/L; multiply by 0.01129; ^6^: MDRD formula. Abbreviations: BP—blood pressure; eGFR—estimated glomerular filtration rate.

## Data Availability

Original data supporting these results are available on request to the corresponding author for reasonable purposes. The data are not publicly available due to privacy.

## Supplementary Materials

The following supporting information can be downloaded at: https://www.mdpi.com/article/10.3390/metabo15010061/s1, Figure S1: Study design and scheme (flow diagram); Figure S2: Schematic illustration of timing of meals, RTD pre-meal beverage, acetaminophen ingestion, and blood sampling protocol; Figure S3: Consort diagram; Figure S4: Glucagon trajectory over 4 h following pre-meal consumption of ultra-concentrated whey protein microgel or placebo to a mixed lunch meal in people with type 2 diabetes mellitus; Figure S5: Trajectories over 2 h following pre-meal consumption of ultra-concentrated whey protein microgel or placebo to a mixed lunch meal in people with type 2 diabetes mellitus of A-GIP; B–CCK; C–PYY; D–Ghrelin; E–Triglycerides; Table S1: Study inclusion and exclusion criteria; Table S2: Pre and post hoc defined analyses; Table S3: C_max_ and T_max_ of biomarkers assessed.
