# Supplementary material for: A Randomized, Placebo-Controlled, Single-Center, Crossover Study to Evaluate the Effects of Pre-Meal Whey Protein Microgel on Post-Prandial Glucometabolic and Amino Acid Response in People with Type 2 Diabetes and Overweight or Obesity
Source: Metabolites. 2025 Jan 16;15(1):61. doi: 10.3390/metabo15010061 (PMC11767963; doi:10.3390/metabo15010061)
Supplement: Supplementary file 1 [file metabolites-15-00061-s001.zip › metabolites-3392300-supplementary.pdf]

Supplementary material

Ian J Neeland, Luiz H de Gregório, Roberto Zagury, Bo Ahrén, Joel Neutel, Christian Darimont, John Corthesy, Yohan Grzywinski, Emilie Perrin, Maximillian von Eynatten, Odd Erik Johansen. A Randomised, Placebo-Controlled, Single-Center, Crossover Study to Evaluate the Effects of Pre-Meal Whey Protein Microgel on Post-Prandial Glucometabolic and Amino-Acid Response in People with Type 2 Diabetes and Overweight or Obesity

**Section - page**	**Description**
Figure S1 - 2	Study design and scheme
Table S1 - 3	Study inclusion and exclusion criteria
Figure S2 - 4	Schematic illustration of timing of meals, RTD pre-meal beverage, acetaminophen ingestion, and blood sampling protocol
Table S2 - 5	Pre- and post-hoc defined analysis
Figure S3 - 6	Consort Diagram
Table S3 - 7	C_{max} and T_{max} of biomarkers assessed
Figure S4 - 8	Glucagon trajectory over 4 hours following pre-meal consumption of ultra-concentrated whey protein microgel or placebo to a mixed lunch meal in people with type 2 diabetes mellitus.
Figure S5 - 9	Trajectories over 2 hours following pre-meal consumption of ultra-concentrated whey protein microgel or placebo to a mixed lunch meal in people with type 2 diabetes mellitus: A- GIP; B – CCK; C – PYY; D – Ghrelin; E - Triglycerides

Figure S1. Study design and scheme

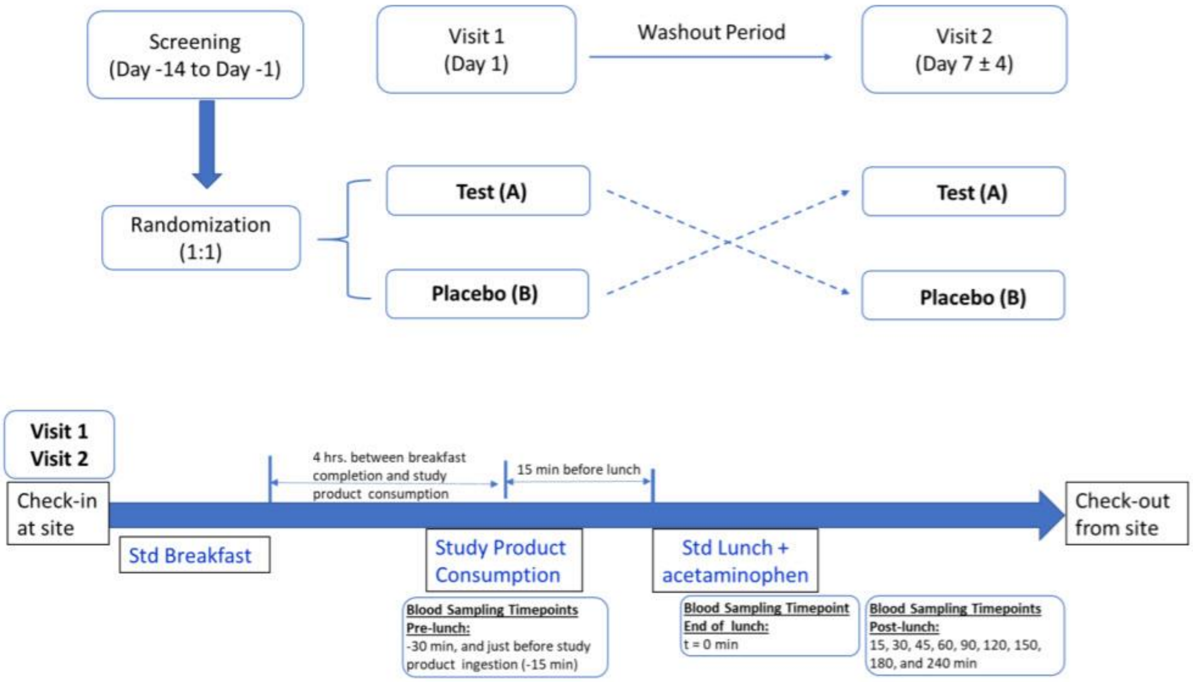

Table S1. Inclusion and exclusion criteria for the study

Inclusion criteria
• Documented type 2 diabetes mellitus (by either HbA1c 6.5 – 10.0 % or a history of type 2 diabetes mellitus diagnosis)<br>• Willing and able to sign written informed consent prior to study entry<br>• Male or female<br>• ≥ 18 years of age<br>• Treatment naïve type 2 diabetes mellitus or on active therapy with metformin at a daily dose of 1000 – 3000 mg at screening (dose of metformin must have been stable for at least 3 months prior to screening)<br>• Haematocrit value greater than or equal to 34.0 % for females and 40 % for males<br>• Haemoglobin value greater than or equal to 11.0 g/dL for females and 13.5 g/dL for males
Exclusion criteria
• Fasting plasma glucose > 220 mg/dl at screening<br>• Impaired kidney function, eGFR < 60 mL/min/1.73 m^2 at screening<br>• BMI > 40 kg/m^2<br>• Elevated liver transaminase > 3 upper limit of normal at screening<br>• Ongoing or recent (i.e. < 3 month) treatment with any oral or injectable glucose-lowering drug other than metformin<br>• Ongoing or recent (i.e. < 3 month) injectable insulin therapy<br>• Ongoing or recent (i.e. < 3 month) weight loss interventions (e.g. dietary weight loss programs) or any history of bariatric surgery or any documented weight loss > 5% within previous 6 months<br>• Ongoing or recent (i.e. < 3 month) treatment with anorectic drugs, systemic steroids, medications known to affect gastric motility, or any condition known to affect gastro-intestinal integrity and food absorption<br>• Major medical/surgical event requiring hospitalization in the last 3 months.<br>• Known allergy and intolerance to product components or acetaminophen<br>• Alcohol intake higher than 2 servings per day (a serving is defined as 0.4 dl of strong alcohols, 1dl of red or white wine, or 3 dl of beer)<br>• Unable to comply with protocol procedures in the opinion of the investigator.<br>• Hierarchical link with the research team members<br>• Positive pregnancy test at screening<br>• Participated in another clinical trial with any investigational drug/new chemical entity within 30 days or 5 half-lives (whichever is longer) prior to screening, or current participation in any investigational trial<br>• Donation of blood or significant amount of blood loss within 8 weeks prior to screening (subjects must also agree to not donate blood within 8 weeks after their last visit).

Figure S2 Schematic illustration of timing of meals, RTD pre-meal beverage, acetaminophen ingestion, and blood sampling protocol

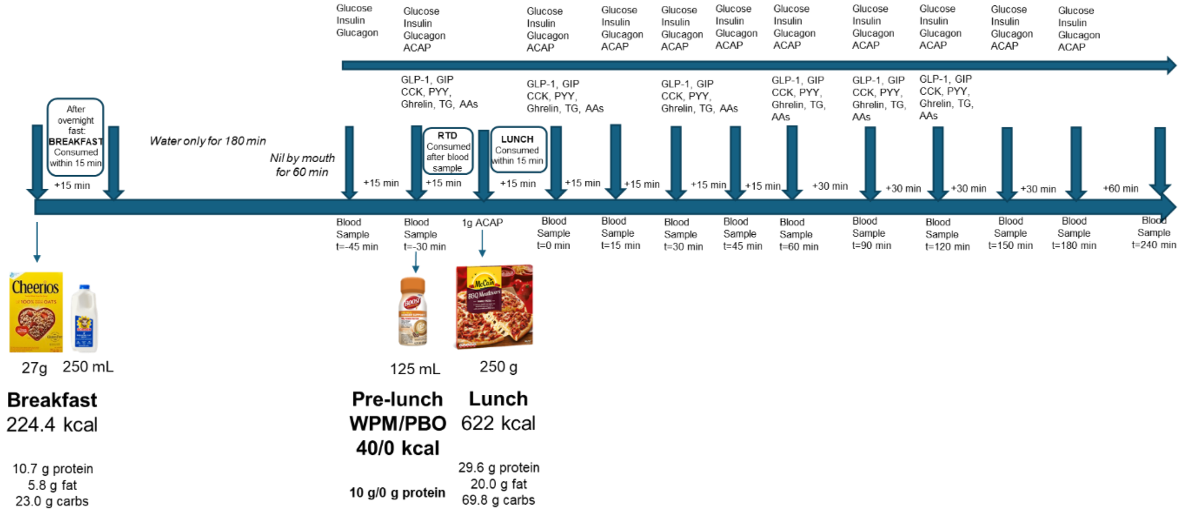

Abbreviations: Min – minutes; t – time; min – minutes; g – gram; AAs – aminoacids; ACAP – acetaminophen; RTD – ready-to-drink; GLP-1 – glucagon like peptide-1; GIP - glucose-dependent insulinotropic polypeptide; CCK – Cholecystokinin; PYY - peptide-YY; TG – triglycerides

Table S2. Pre- and post hoc defined analysis

	Predefined	Post-hoc defined
Glucose	iAUC 0-3h, T_{max}, C_{max}	iAUC 0-2h
Insulin	iAUC 0-3h, T_{max}, C_{max}	iAUC 0-1h
Glucagon	iAUC 0-3h, T_{max}, C_{max}	iAUC 0-1h
GLP-1	iAUC 0-2h, T_{max}, C_{max}	
GIP	iAUC 0-2h, T_{max}, C_{max}	
CCK	iAUC 0-2h, T_{max}, C_{max}	
PYY	iAUC 0-2h, T_{max}, C_{max}	
Ghrelin		Negative iAUC 0-2h<br>Cmin<br>Time to Cmin
Triglycerides	iAUC 0-2h, T_{max}, C_{max}	
Gastric emptying (ACAP)	tAUC 0-4h, T_{max}, C_{max}	tAUC 0-1h
Amino acids		iAUC 0-2h, T_{max}, C_{max}

Abbreviations: ACAP – acetaminophen; GLP-1 – glucagon like peptide-1; GIP - glucose-dependent insulinotropic polypeptide; CCK – Cholecystokinin; PYY - peptide-YY; iAUC – incremental area under the curve; tAUC – total area under the curve

Figure S3. Consort diagram A: placebo; B: Whey protein microgel (test product)

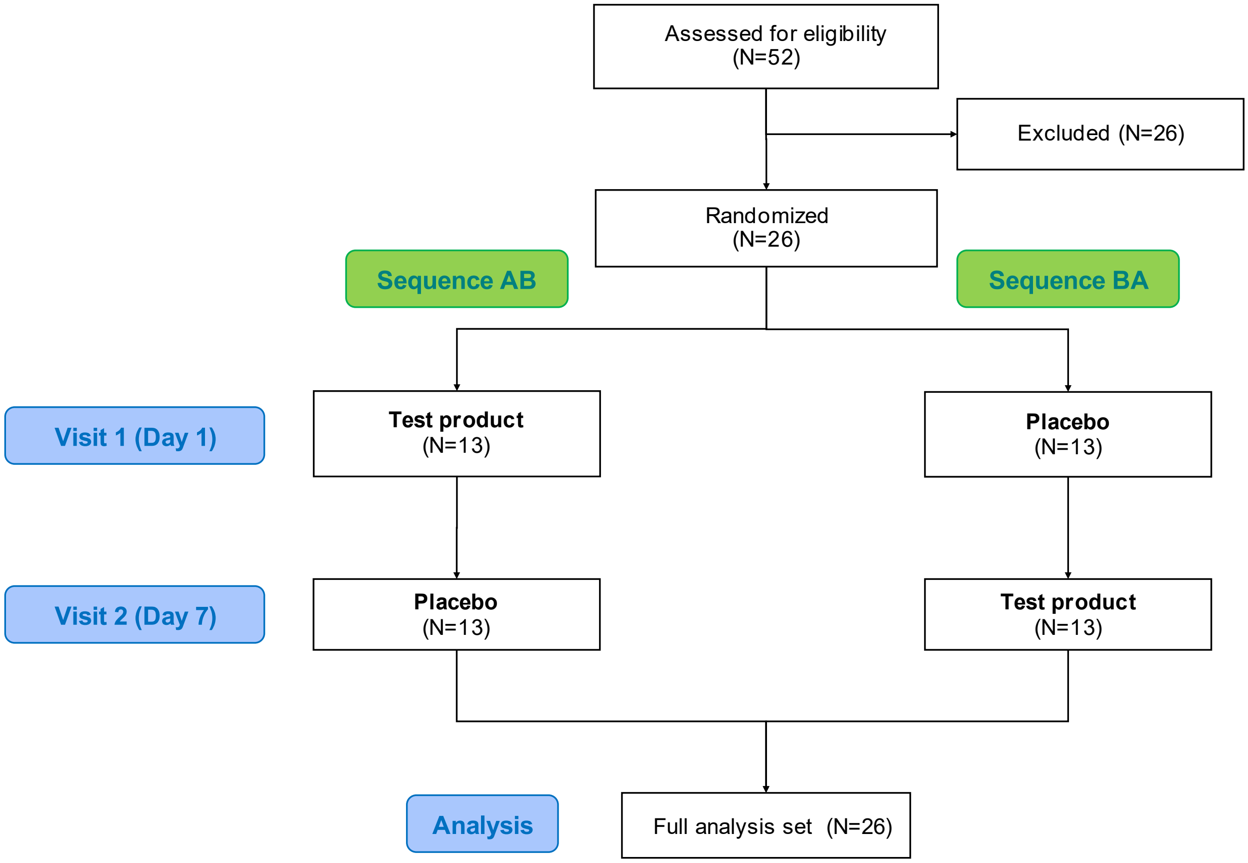

Table S3 C_{max} and T_{max} of the glucometabolic and gastric emptying parameters assessed up to 4 hours in blood for whey protein microgel (WPM) versus placebo. Data given as mean [95% CI].

	**C_{max}**			**T_{max}, min**		
	WPM	Placebo	**Comparison WPM vs Placebo**	WPM	Placebo	**Comparison WPM vs Placebo**
**Glucose, mg/dL**	216 [193, 238]	225 [203, 248]	-10 [-28, 9] *p=0.3019*	104.4 [85.9, 122.9]	59.4 [40.9, 77.9]	45.0 [21.9, 68.1] ***p=0.0005***
**Insulin, µIU/mL**	61.3 [46.2, 76.3]	53.0 [37.9, 68.0]	8.3 [-4.2, 20.8] *p=0.1833*	76.2 [56.9, 95.4]	88.9 [69.6, 108.1]	-12.7 [-40.0, 14.6] *p=0.3541*
**Glucagon, pg/mL**	124.1 [105.7, 142.4]	109.6 [91.2, 127.9]	14.5 [-5.3, 34.3] *p=0.1441*	75.0 [46.7, 103.3]	99.8 [71.5, 128.1]	-24.8 [-64.9, 15.3] *p=0.2194*
**GLP-1, pmol/L**	12.0 [10.6, 13.5]	9.2 [7.7, 10.7]	2.8 [1.1, 4.6] ***p=0.0032***	38.1 [22.5, 53.7]	50.1 [34.2, 66.0]	-12.0 [-29.9, 5.8] p=0.1773
**GIP, pmol/L**	129.3 [109.5. 149.2]	130.7 [110.9, 150.6]	-1.4 [-18.1, 15.3] *p=0.8623*	77.3 [64.4, 90.2]	55.4 [42.5, 68.3]	21.9 [3.7, 40.1] ***p=0.0194***
**PYY, pmol/L**	34.2 [29.7, 38.7]	32.6 [28.2, 37.1]	1.5 [-3.1, 6.2] *p=0.5055*	62.8 [47.0, 78.5]	48.5 [33.0, 63.9]	14.3 [-6.4, 35.1] *p=0.1662*
**CCK, pg/mL**	157.81 [81.6, 234.0]	144.2 [68.0, 220.4]	13.6 [-16.5, 43.7] *p=0.3601*	51.9 [35.0, 68.9]	43.9 [26.9, 60.8]	8.1 [-15.9, 32.0] *p=0.5014*
**Ghrelin, pg/mL#**	305.7 [284.1, 327.4]	307.9 [287.1, 328.8]	-2.2 [-22.1, 17.6] *p=0.8122*	48.4 [25.2, 71.6]	56.0 [34.5, 77.4]	-7.6 [-39.0, 23.7] *p=0.6119*
**Triglycerides, mg/dL**	221 [184, 258]	218 [181, 256]	3 [-29, 34] *p=0.8718*	87.7 [70.0, 105.4]	85.5 [67.4, 103.5]	2.2 [-18.1, 22.6] *p=0.8217*
**Acet-aminophen, ng/mL**	11719 [10064, 13374]	14156 [12501, 15812]	-2437 [-4271, -603] ***p=0.0113***	29.4 [18.3, 41.0]	24.8 [13.7, 35.9]	4.6 [-8.8, 18.0] *p=0.4840*

#C_{min} and T_{min} in stead of C_{max} and T_{max} is calculated.

Abbreviations: Min – minutes; GLP-1 – glucagon like peptide-1; GIP - glucose-dependent insulinotropic polypeptide; CCK – Cholecystokinin; PYY - peptide-YY

Figure S4 Glucagon trajectory over 4 hours following pre-meal consumption of ultra-concentrated whey protein microgel or placebo to a mixed lunch meal in people with type 2 diabetes mellitus.

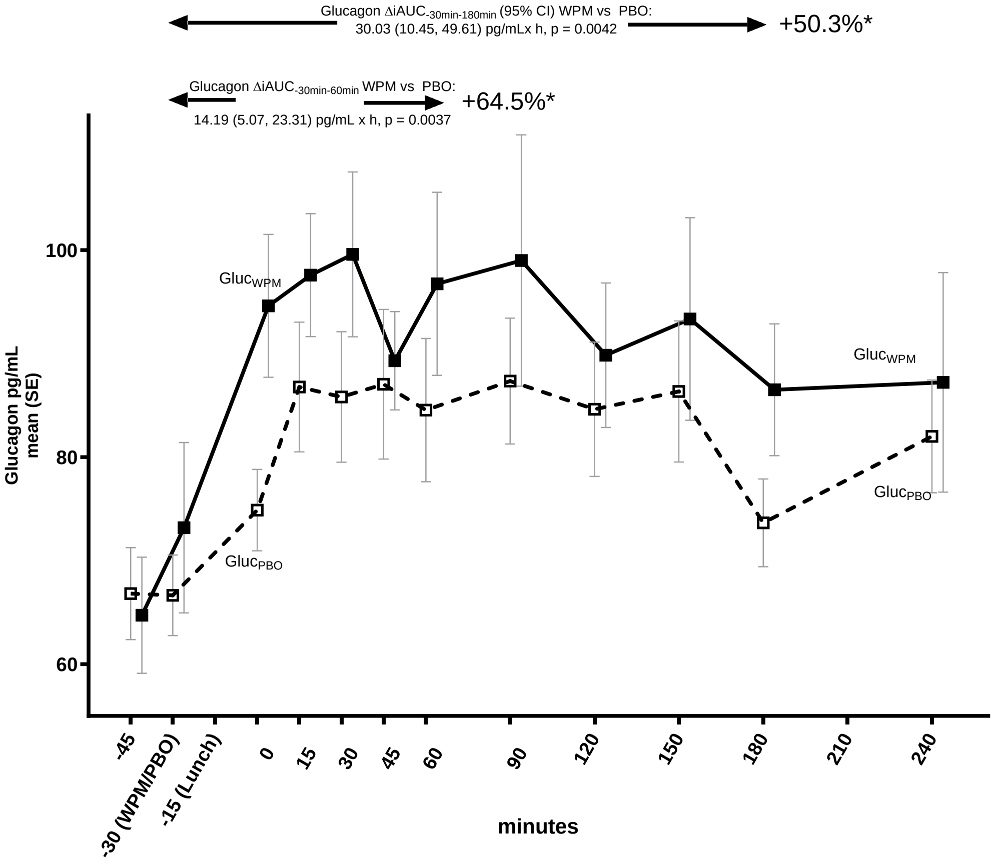

Abbreviations: iAUC – incremental area under the curve, PBO – placebo, WPM, whey protein microgel, SE – standard error, h - hours.
*: p<0.05.

Figure S5 Trajectories of (A) Glucose-dependent insulinotropic polypeptide (GIP), (B) Cholecystokinin, (C) peptide YY (PYY), (D) Ghrelin, and (E) Triglycerides, over 2 hours following pre-meal consumption of ultra-concentrated whey protein microgel or placebo to a mixed lunch meal in people with type 2 diabetes mellitus.

A GIP

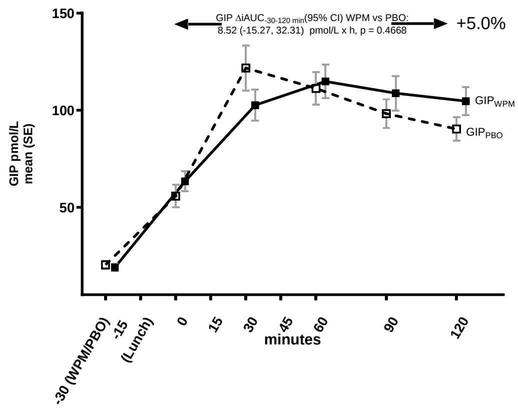

B CCK

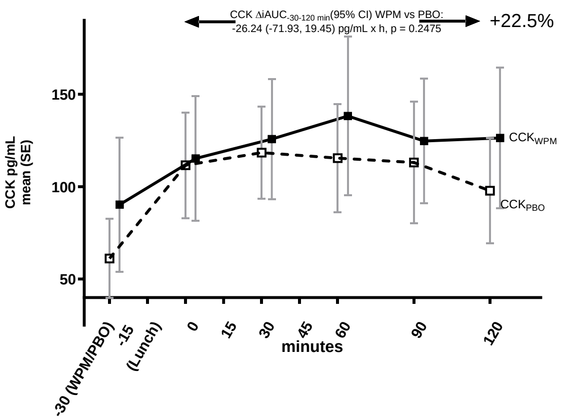

C PYY

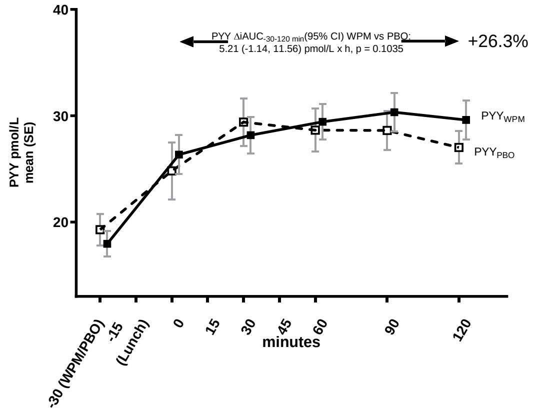

D Ghrelin

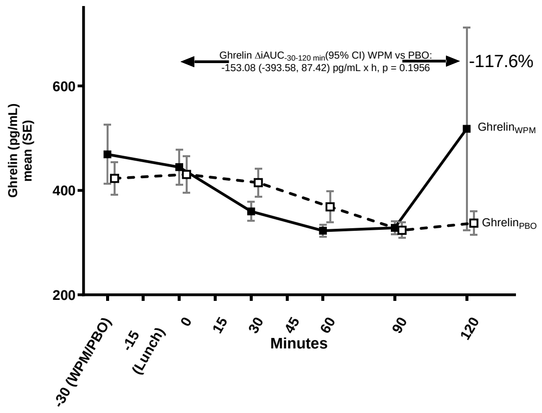

E Triglycerides

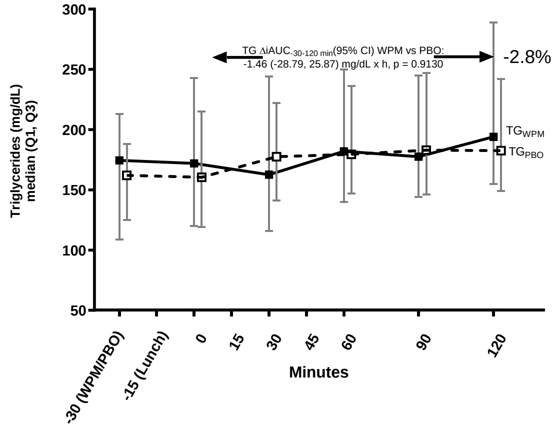

Abbreviations: GIP - glucose-dependent insulinotropic polypeptide, CCK – Cholecystokinin, PYY – peptide YY, TG – triglycerides, iAUC – incremental area under the curve, PBO – placebo, WPM, whey protein microgel, SE – standard error, h – hours, Q1 – first quartile, Q3 – third quartile
